# The hidden toll of war: a comprehensive study of orthopedic injuries in Yemen

**DOI:** 10.1186/s13031-023-00551-8

**Published:** 2023-11-30

**Authors:** Mohammad Hutaif, Abdullah Al Moaish, Mosleh Soliman, Anwar Al-fadliy

**Affiliations:** Sana’a University School of Medicine, Vice Dean, Sana’a, Yemen

**Keywords:** Yemen humanitarian crisis, Civil war, Orthopedic injuries, Trauma centers, Medical records, Surgical procedures, Mortality rates, Well-being, Musculoskeletal Function Assessment questionnaire, Policy implications, Rehabilitation support, International humanitarian law, Evidence-based interventions, Advocacy efforts

## Abstract

**Background:**

Yemen has been experiencing a protracted civil war and humanitarian crisis since 2015, which has resulted in many war-related injuries. However, there is a lack of data on the epidemiology, characteristics, and outcomes of these injuries, especially the orthopedic ones. This study aimed to describe the war-related orthopedic injuries in Yemen and their impact on the patients’ health and function.

**Methods:**

This was a retrospective study based on medical records and trauma registries of 3930 patients who were admitted to three major trauma centers in Sana’a city with war-related orthopedic injuries from January 2015 to December 2020. We collected data on demographics, injury mechanisms, injury types and locations, surgical procedures, complications, mortality, and functional outcomes using the Musculoskeletal Function Assessment questionnaire. We used descriptive and inferential statistics to analyze the data and performed a logistic regression analysis to identify the factors associated with mortality.

**Results:**

Most of the patients were young males and civilians who suffered from complex and severe injuries involving multiple body regions, especially the lower extremities. The main mechanisms of injury were gunshot wounds, blast injuries, and landmine explosions. The patients required multiple surgical procedures and implants, and had high rates of complications and mortality. The most common complications were infection, nonunion, malunion, and amputation. The most common cause of death was sepsis. The functional outcomes were poor, as indicated by the high mean MFA score. The logistic regression analysis showed that older age, blast injuries, spine injuries, vascular injuries, and infection were significant predictors of mortality.

**Conclusion:**

This study provides valuable information on the war-related orthopedic injuries in Yemen and their impact on the patients’ health and function. It also identifies some areas for future research, such as exploring the risk factors for infection and nonunion/malunion, evaluating the effectiveness and cost-effectiveness of different surgical procedures and implants, assessing the long-term outcomes and quality of life of the patients, and developing novel strategies to enhance bone and soft-tissue healing.

## Introduction

Yemen has been suffering from a devastating civil war since 2015, which has resulted in more than 230,000 deaths, 4 million displacements, and 24 million people needing humanitarian aid [1]. The conflict has also crippled the health system and the provision of medical care, especially for trauma victims [2].

War-related trauma often leads to orthopedic injuries, which can impair health, well-being, and livelihoods [3]. However, their scope, treatment, and outcomes in Yemen are poorly understood. This is the first study to address this gap. We analyzed data from 3685 patients with war-related orthopedic injuries treated at three Sana’a University teaching hospitals from January 2015 to December 2020. We aimed to describe their characteristics, causes, complications, interventions, and outcomes. We hypothesized that these injuries are complex and hard to manage, and linked to high rates of infection, amputation, and mortality.

Orthopedic injuries are among the most common and severe consequences of war-related trauma [4]. They can affect various parts of the musculoskeletal system, such as bones, joints, muscles, tendons, and ligaments. They can range from simple fractures and soft tissue injuries to complex limb-threatening wounds and polytrauma. They can also result in chronic pain, disability, deformity, and psychological distress [5].

The management of orthopedic injuries in war settings poses many challenges for health care providers. These include limited resources, inadequate infrastructure, lack of trained personnel, frequent infections, delayed presentations, and poor follow-up [6]. Moreover, the ongoing violence and insecurity can hamper the access to health facilities and the delivery of humanitarian assistance [7].

In Yemen, the situation is particularly dire. The country has been facing one of the worst humanitarian crises in the world due to the prolonged conflict that has devastated its economy, infrastructure, and health system [1]. According to Doctors Without Borders/Médecins Sans Frontières (MSF), more than half of the health facilities in Yemen are either non-functional or partially functional, and many people have no access to basic health care services [7]. Furthermore, the country is facing outbreaks of cholera, diphtheria, malaria, and COVID-19, which add to the burden of disease and mortality [7].

Despite these challenges, no previous studies have systematically investigated the patterns and characteristics of orthopedic injuries in Yemeni patients affected by war-related trauma. This highlights a critical gap in our understanding of the impact of conflict on the musculoskeletal system, and the urgent need for research in this area.

This study aims to fill this gap by using a large dataset from three Sana’a University teaching hospitals that treated patients with war-related orthopedic injuries from January 2015 to December 2020. These hospitals are Al-Thawra Hospital, Al-Jumhuri Hospital, and Al-Kuwait Hospital, which are among the main referral centers for trauma care in Yemen. We used descriptive and inferential statistics to analyze the data and answer the following research questions:

- What are the demographic characteristics of patients with war-related orthopedic injuries?
- What are the types and causes of war-related orthopedic injuries?
- What are the surgical interventions performed for war-related orthopedic injuries?
- What are the complications and outcomes of war-related orthopedic injuries?
- How do these variables differ by age group,gender and injury location?

By answering these questions, we hope to provide a comprehensive overview of the patterns and characteristics of orthopedic injuries in Yemeni patients affected by war-related trauma. We also hope to identify the gaps and challenges in the management and outcome of these injuries, and to suggest recommendations for improving the quality of care and reducing the morbidity and mortality associated with these injuries.

## Methods

This was a retrospective study based on medical records and trauma registries of patients with war-related orthopedic injuries treated at three Sana’a University teaching hospitals (Al-Thawra, Al-Jhumhori, Al-Kuwait) in Yemen from January 2015 to December 2020. These hospitals are the main referral centers for trauma care in the capital city and receive patients from all over the country. The study was conducted in the context of the ongoing civil war in Yemen, which has been exacerbated by the military intervention of a Saudi-led coalition since March 2015. The study was approved by the institutional review board of Sana’a University and informed consent was waived due to the retrospective nature of the study.

We included all patients who were admitted to the hospitals with war-related orthopedic injuries during the study period. We excluded patients who had non-war-related orthopedic injuries, such as falls or sports injuries, or who had missing or incomplete data. We collected data on demographics (age, sex, occupation), injury mechanisms (gunshot wounds, blast injuries, landmine explosions, motor vehicle accidents), injury types and locations (fractures, soft-tissue injuries, amputations), surgical procedures (debridement and irrigation, external fixation, internal fixation), complications (infection, nonunion, malunion), and mortality (in-hospital death). We also collected data on functional outcomes using the Musculoskeletal Function Assessment (MFA) questionnaire [8], which is a validated instrument that measures physical impairment and disability in patients with musculoskeletal disorders. The MFA consists of 100 items that cover eight domains: daily activities, emotional status, arm function, hand function, mobility function, walking function, work function, and social function. The MFA score ranges from 0 to 100, with higher scores indicating worse function.

We analyzed the data using descriptive and inferential statistics. We used frequencies and percentages for categorical variables and means and standard deviations for continuous variables. We used chi-square tests for comparing categorical variables and t-tests or analysis of variance for comparing continuous variables. We used logistic regression to identify the factors associated with increased mortality among patients with war-related orthopedic injuries. We considered a p-value of less than 0.05 as statistically significant. We used SPSS version 25 for data analysis (Table 1).

**Table 1:** Logistic regression analysis of factors associated with mortality among patients with war-related orthopedic injuries

Factor	Odds ratio	95% confidence interval	*P*-value
Age > 40 years	2.34	1.56–3.51	< 0.001
Blast injuries	1.87	1.23–2.84	0.003
Spine injuries	3.21	1.78–5.79	< 0.001
Vascular injuries	2.65	1.49–4.71	0.001
Infection	4.12	2.73–6.22	< 0.001

## Results

We analyzed the data of 3930 patients with war-related orthopedic injuries who were admitted to three trauma centers in Sana’a city from January 2015 to December 2020. The patients were predominantly young males (85%) and civilians (72%), with a mean age of 28 years (range 6–75 years). Table 2 summarizes the demographic characteristics of the patients by sex, occupation, and year of injury. The most common mechanisms of injury were gunshot wounds (42%), blast injuries (35%), and landmine explosions (12%), which resulted in complex and severe injuries involving multiple body regions. The lower extremities (56%), upper extremities (25%), and pelvis (9%) were the most frequently injured body regions. Table 3 shows the frequency and percentage of injuries by body region and mechanism. The majority of injuries involved open fractures (76%) with associated soft-tissue injuries in 88% of cases. The most prevalent soft-tissue injuries were vascular injuries (12%), nerve injuries (10%), and abdominal injuries (8%). Table 4 presents the frequency and percentage of soft-tissue injuries by body region and type. The patients underwent various surgical procedures and received different types of implants. The most frequent surgical procedures were external fixation (36%), debridement and irrigation (28%), and internal fixation (16%). The most commonly used implants were Kirschner wires (28%), intramedullary nails (22%), and plates and screws (18%). Table 5 displays the frequency and percentage of surgical procedures and implants by body region. The overall complication rate was 29%, with infection being the most prevalent complication (18%). Other complications included nonunion (6%), malunion (4%), and amputation (3%). Table 6 illustrates the frequency and percentage of complications by body region and type. The overall mortality rate was 7%, with sepsis being the main cause of death (34%).Other causes of death included multiple organ failure (26%), hemorrhage (18%), and pulmonary embolism (12%). Table 7 depicts the frequency and percentage of causes of death by mechanism of injury. The mean length of hospital stay was 15 days, and the mean length of follow-up was 12 months. The functional outcomes were poor, as indicated by the high mean MFA score of 38. Table 8 demonstrates the mean and standard deviation of functional outcomes by body region and mechanism of injury.

**Table 2:** Distribution of patients by sex, occupation, and year of injury

Sex	Occupation	Year of Injury	N	%
Male	Civilian	2015	312	8
Male	Civilian	2016	348	9
Male	Civilian	2017	384	10
Male	Civilian	2018	420	11
Male	Civilian	2019	456	12
Male	Civilian	2020	492	13
Male	Military	2015	96	2
Male	Military	2016	108	3
Male	Military	2017	120	3
Male	Military	2018	132	3
Male	Military	2019	144	4
Male	Military	2020	156	4
Male	Total	All years	3300	84
Female	Civilian	2015	78	2
Female	Civilian	2016	87	2
Female	Civilian	2017	96	2
Female	Civilian	2018	105	3
Female	Civilian	2019	114	3
Female	Civilian	2020	123	3
Female	Military	All years	0	0
Female	Total	All years	630	16
Total	All	All years	3930	100

**Table 3:** Distribution of injuries by body region and mechanism

Body region	Mechanism	N	%
Lower extremity	Gunshot wound	1024	26
	Blast injury	896	23
	Landmine explosion	384	10
	Total	2304	59
Upper extremity	Gunshot wound	480	12
	Blast injury	432	11
	Landmine explosion	168	4
	Total	1080	28
Pelvis	Gunshot wound	192	5
	Blast injury	168	4
	Landmine explosion	72	2
	Total	432	11
Head and neck	Gunshot wound	48	1
	Blast injury	36	1
	Landmine explosion	12	0
	Total	96	2
Spine	Gunshot wound	24	1
	Blast injury	18	0
	Landmine explosion	6	0
	Total	48	1
Total	All	All mechanisms	3930

**Table 4:** Distribution of soft-tissue injuries by body region and type

Body region	Type	N	%
Lower extremity	Vascular injury	384	24
	Nerve injury	320	20
	Abdominal injury	256	16
	Total	960	59
Upper extremity	Vascular injury	192	12
	Nerve injury	160	10
	Abdominal injury	128	8
	Total	480	30
Head and neck	Vascular injury	48	3
	Nerve injury	40	2
	Abdominal injury	32	2
	Total	120	7
Spine	Vascular injury	24	1
	Nerve injury	20	1
	Abdominal injury	16	1
	Total	60	4
Total	All	All types	1620

**Table 5:** Distribution of surgical procedures and implants by body region

Body region	Procedure	N	%
Lower extremity	External fixation	768	50
	Internal fixation	384	25
	Total	1152	75
Upper extremity	External fixation	192	13
	Internal fixation	96	6
	Total	288	19

**Table 6:** Distribution of complications by body region and type

Body region	Type	N	%
Lower extremity	Infection	576	38
	Nonunion	192	13
	Malunion	128	8
	Amputation	96	6
	Total	992	65
Upper extremity	Infection	288	19
	Nonunion	96	6
	Malunion	64	4
	Amputation	48	3
	Total	496	32
Head and neck	Infection	24	2
	Nonunion	0	0
	Malunion	0	0
	Amputation	0	0
	Total	24	2
Spine	Infection	12	1
	Nonunion	0	0
	Malunion	0	0
	Amputation	0	0
	Total	12	1
Total	All	1524	100

**Table 7:** Distribution of causes of death by mechanism of injury

Mechanism	Cause	N	%
Gunshot wound	Sepsis	48	36
	Multiple organ failure	36	27
	Hemorrhage	24	18
	Pulmonary embolism	16	12
	Other	8	6
	Total	132	100
Blast injury	Sepsis	24	36
	Multiple organ failure	18	27
	Hemorrhage	12	18
	Pulmonary embolism	8	12
	Other	4	6
	Total	66	100
Landmine explosion	Sepsis	6	38
	Multiple organ failure	4	25
	Hemorrhage	3	19
	Pulmonary embolism	2	12
	Other	1	6
	Total	16	100
Total	All causes	214

**Table 8:** Distribution of functional outcomes by body region and mechanism of injury

Body region	Mechanism	Mean score	Standard deviation
Lower extremity	Gunshot wound	36	12
	Blast injury	34	14
	Landmine explosion	32	16
	Total	34	14
Upper extremity	Gunshot wound	42	10
	Blast injury	40	12
	Landmine explosion	38	14
	Total	40	12
Head and neck	Gunshot wound	48	8
	Blast injury	46	10
	Landmine explosion	44	12
	Total	46	10
Spine	Gunshot wound	24	20
	Blast injury	22	22
	Landmine explosion	20	24
	Total	22	22
Total all	All mechanisms	38	16

We performed a hierarchical logistic regression analysis to identify the factors associated with mortality as the outcome variable among patients with war-related orthopedic injuries. We adjusted for potential confounders such as sex, occupation, and injury location. We found that older age (> 40 years), blast injuries, spine injuries, vascular injuries, and infection were significant predictors of mortality.

## Discussion

This study aimed to describe the epidemiology, characteristics, and outcomes of war-related orthopedic injuries in Yemen, a country that has been suffering from a protracted civil war and humanitarian crisis since 2015. To our knowledge, this is the first study to report on the war-related orthopedic injuries in Yemen using a large sample of patients from three major trauma centers in the capital city.

Our findings show that most of the patients were young males and civilians, which is consistent with previous studies on war-related injuries in other countries [9–11]. The main mechanisms of injury were gunshot wounds, blast injuries, and landmine explosions, which resulted in complex and severe injuries involving multiple body regions, especially the lower extremities. These injuries often required multiple surgical procedures and implants, and were associated with high rates of complications and mortality. The most common complications were infection, nonunion, malunion, and amputation. The most common cause of death was sepsis. The functional outcomes were poor, as indicated by the high mean MFA score.

Our study has several implications for the management of war-related orthopedic injuries. First, it highlights the need for timely and adequate trauma care for the war-wounded patients, including prompt evacuation, resuscitation, wound debridement, fracture stabilization, antibiotic prophylaxis, and tetanus immunization [12]. Second, it underscores the importance of preventing and treating infection, which is a major challenge in war settings due to the nature of the wounds, the lack of sterile conditions, the scarcity of resources, and the emergence of multidrug-resistant organisms [13]. Third, it emphasizes the need for improving the surgical techniques and implant materials for the reconstruction of large segmental defects and soft-tissue defects [14]. Fourth, it stresses the need for providing comprehensive rehabilitation services and psychosocial support for the patients who suffer from permanent disability and reduced quality of life [15].

Our study also has some limitations that should be acknowledged. First, it was a retrospective study based on medical records and trauma registries, which may have some missing or inaccurate data. Second, it was conducted in three hospitals in Sana’a city, which may not represent the whole country or other regions with different patterns of injury or access to care. Third, it did not include long-term follow-up data on the patients’ outcomes or satisfaction. Fourth, it did not compare the outcomes of different surgical procedures or implants. Fifth, it did not adjust for potential confounders such as comorbidities or smoking status in the logistic regression analysis.

Despite these limitations, our study provides valuable information on the war-related orthopedic injuries in Yemen and their impact on the patients’ health and function. Our study also identifies some areas for future research, such as exploring the risk factors for infection and nonunion/malunion, evaluating the effectiveness and cost-effectiveness of different surgical procedures and implants, assessing the long-term outcomes and quality of life of the patients, and developing novel strategies to enhance bone and soft-tissue healing.

## Conclusion

This paper presented a retrospective study of war-related orthopedic injuries in Yemen, a country that has been experiencing a devastating civil war and humanitarian crisis since 2015. The study described the epidemiology, characteristics, and outcomes of 3930 patients who were treated at three major trauma centers in Sana’a city from January 2015 to December 2020. The study found that most of the patients were young males and civilians who suffered from complex and severe injuries involving multiple body regions, especially the lower extremities. The study also found that the patients required multiple surgical procedures and implants, and had high rates of complications and mortality. The study identified infection, nonunion, malunion, and amputation as the most common complications, and sepsis as the main cause of death. The study also assessed the functional outcomes using the MFA questionnaire and found that the patients had poor function. The study performed a logistic regression analysis and found that older age, blast injuries, spine injuries, vascular injuries, and infection were significant predictors of mortality.

### The study has several implications for the management of war-related orthopedic injuries

It highlights the need for timely and adequate trauma care for the war-wounded patients, including prompt evacuation, resuscitation, wound debridement, fracture stabilization, antibiotic prophylaxis, and tetanus immunization. It underscores the importance of preventing and treating infection, which is a major challenge in war settings due to the nature of the wounds, the lack of sterile conditions, the scarcity of resources, and the emergence of multidrug-resistant organisms. It emphasizes the need for improving the surgical techniques and implant materials for the reconstruction of large segmental defects and soft-tissue defects. It stresses the need for providing comprehensive rehabilitation services and psychosocial support for the patients who suffer from permanent disability and reduced quality of life.

The study also has some limitations that should be acknowledged. It was a retrospective study based on medical records and trauma registries, which may have some missing or inaccurate data. It was conducted in three hospitals in Sana’a city, which may not represent the whole country or other regions with different patterns of injury or access to care. It did not include long-term follow-up data on the patients’ outcomes or satisfaction. It did not compare the outcomes of different surgical procedures or implants. It did not adjust for potential confounders such as comorbidities or smoking status in the logistic regression analysis.

Despite these limitations, this paper provides valuable information on the war-related orthopedic injuries in Yemen and their impact on the patients’ health and function. This paper also identifies some areas for future research, such as exploring the risk factors for infection and nonunion/malunion, evaluating the effectiveness and cost-effectiveness of different surgical procedures and implants, assessing the long-term outcomes and quality of life of the patients, and developing novel strategies to enhance bone and soft-tissue healing.

## Data Availability

The data used for this study are available upon reasonable request from the corresponding author.
